# Science on the Rise in Developing Countries

**DOI:** 10.1371/journal.pbio.0020001

**Published:** 2004-01-20

**Authors:** Milena Holmgren, Stefan A Schnitzer

## Abstract

The disparity in the scientific output between developed and developing counties is dramatic, but, as the Americas show, this grim picture is improving

Kofi Annan, the Secretary-General of the United Nations, recently called attention to the clear inequalities in science between developing and developed countries and to the challenges of building bridges across these gaps that should bring the United Nations and the world scientific community closer to each other (Annan 2003). Mr. Annan stressed the importance of reducing the inequalities in science between developed and developing countries, asserting that “This unbalanced distribution of scientific activity generates serious problems not only for the scientific community in the developing countries, but for development itself.” Indeed, Mr. Annan's sentiments have also been echoed recently by several scientists, who present overwhelming evidence for the disparity in scientific output between the developing and already developed countries (Gibbs 1995; May 1997; Goldemberg 1998; Riddoch 2000). For example, recent United Nations Educational, Scientific, and Cultural Organization (UNESCO) estimates (UNESCO 2001) indicate that, in 1997, the developed countries accounted for some 84% of the global investment in scientific research and development, had approximately 72% of the world researchers, and produced approximately 88% of all scientific and technical publications registered by the Science Citation Index (SCI). North America and Europe clearly dominate the number of scientific publications produced annually, with 36.6% and 37.5%, respectively, worldwide (UNESCO 2001).

*North America and Europe clearly dominate the number of scientific publications produced annually.*

It is rather obvious that richer countries are able to invest more resources in science and therefore account for the largest number of publications. It is also likely that there is a statistical bias on the part of the SCI as a bibliometric database, since it represents North American and European publications far better than those of the rest of the world (Gibbs 1995; May 1997; Alonso and Fernández-Juricic 2001; Vohora and Vohora 2001). But is the disparity in scientific contributions between the developed and developing worlds actually remaining unchanged or even increasing, as Mr. Annan has implied? A closer look at the trends over the last decade reveals important advances in developing countries. For example, Latin America and China, although representing, respectively, only 1.8% and 2% of scientific publications worldwide, have increased the number of their publications between 1990 and 1997 by 36% and 70%, respectively, which is a much higher percentage than the increments reached by Europe (10%) and industrial Asia (26%). The percentage of global scientific publications from North America actually decreased by 8% over the same period (UNESCO 2001).

## Publishing Trends in the Americas

Using the SCI databases produced by the Institute for Scientific Information (ISI), as well as data compiled by the Red Iberoamericana de Indicadores de Ciencia y Tecnología (RICYT), we examined the differences in the number and proportion of scientific publications between the developed world and the developing world from 1990 until 2000, focusing on the Americas as a case study. Not surprisingly, there was a huge disparity in the number of publications from 1990 until 2000, with the United States contributing the lion's share (84.2%), followed by Canada (10.35%). Latin America as a whole contributed only 5.45% to the total number of scientific publications in these ten years (RICYT 2002).

**Figure 1:**
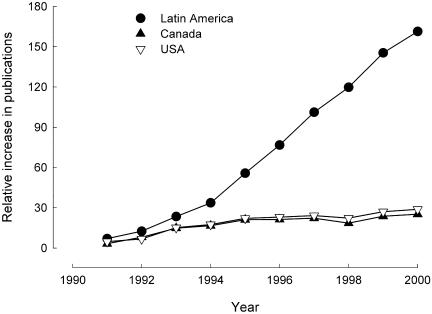
Relative Increase in Scientific Publications in the Americas This figure shows the relative increase in publication in the Americas measured as the proportional change (%) in the number of SCI publications compared with the number of publications in 1990 (RICYT 2002).

**Figure 2:**
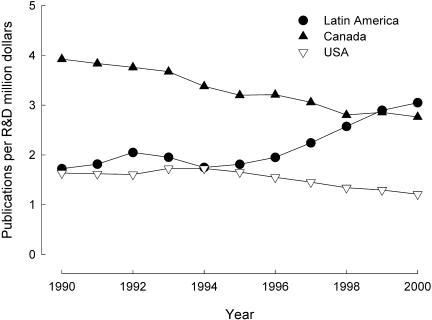
Number of SCI Publications per Million Dollars This figure shows the number of SCI publications per million dollars that are invested in research and development in the Americas (RICYT 2002).

The total number of publications, however, is not necessarily the best measure for assessing scientific productivity or technical advances (May 1997). More relevant measurements for these factors include the proportional change in the number of publications and the total number of publications when corrected for investment in research and development (May 1997). The proportional change in the number of publications, using 1990 as a comparison, revealed that scientific publishing in Latin America increased the most rapidly in the Americas, far outpacing the United States and Canada (Figure 1). Further analyses, correcting the number of overall publications for the amount of money invested in research and development for each region, also show that, in contrast to both Canada and United States, the trend in Latin America has been an increase in relative output throughout the 1990s (Figure 2). Moreover, when taking into account the amount of research money available to researchers, Latin America actually out-published the United States and Canada by the year 2000 (Figure 2). Although the cost of research is undoubtedly cheaper in the developing world due to relatively low researcher salaries, overhead and other work standards, these factors do not explain the substantial increase in the number of publications per amount of money allocated to research and development in Latin America, particularly from 1995 until 2000 (Figure 2).

Other relative indicators of scientific productivity, such as the number of publications picked up by the SCI in relation to the number of scientists in a particular country, also demonstrate that such developing regions as Latin America are making substantial contributions to science, despite the fact that the average proportion of gross domestic product (GDP) invested in science in Latin America throughout this 10-year period was only 21% of the amount invested in United States (RICYT 2002). Indeed, this scientific productivity is remarkable when we compare it with the relatively low investment in science itself as compared with the GDP of Latin America as a whole. In fact, Albornoz (2001) concluded that, as a group, Latin America could afford to invest a much higher proportion of its resources in scientific research and development. Latin American investment in research and development represented only 0.59% of the regional GDP in 1998, a very weak effort compared with that of the United States (2.84%) and Canada (1.5%).

Among Latin American countries, there is a high degree of variability in publication rate as well as in financial investment in science and technology. Some countries have performed particularly well. For example, Uruguay, Chile, Panama, and Cuba averaged, respectively, 6.8, 5.3, 5.2, and 3.4 publications per million dollars of research and development investment in the 10 years studied, which is notoriously high compared with United States (1.5) and even Canada (3.3) (RICYT 2002). Other countries, such as Costa Rica, Cuba, Brazil, and Chile, have invested a much greater proportion of their GDP in research and development than the other countries of this region (Albornoz 2001).

*Why has the number of publications per dollar invested in research and development been increasing in Latin America while decreasing in United States and Canada?*

## Explaining the Increase in Publishing Productivity in Latin America

One potential explanation for the increase in scientific productivity in Latin America is that scientific development during the 1990s was particularly strong for many countries of this region. Indeed, this would explain the rapid rise in the number of publications in Latin America compared with the relatively flat increases in the United States and Canada, which were publishing just as well at the beginning of the decade. A potentially more important question, however, is why the number of publications per dollar invested in research and development has been increasing in Latin America while decreasing in the United States and Canada. This pattern could be the result of a variety of factors, none of which are mutually exclusive. It is possible that publishing in international journals as a measure of scientific productivity is becoming more important in Latin America. Increased funding to the most productive scientists from the national science development programs might have been an important stimulus. International cooperation resulting in more scientific collaborations among scientists in Latin America, Europe, and the United States may also have increased the relative number of publications in Latin America. In contrast, the decreasing trends in the number of publications per investment dollar in Canada and United States could reflect a trend towards more costly research in larger scientific programs.

## Scientific Impact from Latin America

What, exactly, is the relative impact of such developing regions as Latin America on the scientific community? We used SCI 2001 data to examine the proportion of publications in the area of ecology (including the fields of evolutionary biology, conservation biology, and global change biology) between 1990 and 2002 in both the two top general science journals (*Nature* and *Science*; with impact factors of 27.96 and 23.33, respectively) and in the 20 top ecological journals (with impact factors of 10.51–2.47) (ISI 2001a). We credited a region with a publication if any of the authors were affiliated with institutions from that region. Thus, more than one region would receive credit for a single publication if that publication had been written by multiple authors from institutions of different regions.

For the top 20 ecological journals, the American subcontinents of South, Central, and North America accounted for 62% of the publications worldwide. Within the Americas, however, Latin America represented only 6%, while Canada and United States accounted, respectively, for 13% and 82% of the top 20 ecological publications. When we examined the data as contributions to the top 10 ecological journals (impact factors 10.51–3.31) versus the top 11–20 (impact factors 3.28–2.47), the Latin American countries contributed nearly twice as many publications to journals in the second category (8% in the top 11–20 compared with 4% in the top 10). These findings suggest that publications from such developing regions as Latin America are falling short of reaching the top journals. In contrast, the United States contributed somewhat more publications to the top 10 journals (84%) than the top 11–20 journals (79%). The difference in the proportion of publications contributed by the United States to the top 10 and top 20 journals was even more pronounced when we examined it in respect to worldwide publications. In this case, the United States contributed 60% of the publications to the top 10 journals and only 40% of the publications to the top 11–20 journals.

Interestingly, the proportion of publications from Latin America, the United States, and Canada across all subject areas in *Science* and *Nature* were nearly identical to those of the top 20 ecological journals. In *Science* and *Nature*, Latin America had 7% of the publications within the Americas versus 6% in the top 20 ecological journals, whereas the United States and Canada had 81% versus 82% and 12% versus 13%, respectively. These similarities suggest that the Latin American researchers are not shying away from the two top-ranked general science journals. However, publishing in *Science* and *Nature* was not enough to gain prominence, as evidenced by the number of citations of these researchers. The latest list of the 247 most-cited researchers in ecology and environmental sciences emphasizes the overwhelming contributions of authors from North America (73%) and Europe (21%) (ISI 2001b). No researcher working in a Latin American institution was included in the remaining 6%. Overall, these data indicate that the scientific output in the field of ecology in Latin America is having a relatively low impact in the international scientific community and is underrepresented in the top international journals, despite its robust productivity as measured by the number of publications per researcher funding amount. Similar findings were also reported for Asia (Swinbanks et al. 1997) and thus could be a general phenomenon in the developing world.

Although there are outstanding scientific researchers in the developing world who independently are making important contributions to the international scientific community, they are the exception. Why, in general, do Latin American scientists often fail to reach the top journals or become amongst the most cited researchers in their fields? One possibility is that the main research agendas between both regions are somewhat different and that the top journals, which are published in the developed world, respond more to the scientific mainstream of the developed regions. This is not to suggest any sort of conspiracy, but rather it implies that the perception of the most important science is linked to the region and that because the major funding agencies as well as most prominent journals share a similar economic region, they also share the same perception of what science is most interesting to them. Another consideration is that more local journals from developed regions are listed by the SCI than similar journals from developing regions (Gibbs 1995). Consequently, there are more high-profile regional publication opportunities available to scientists from the developed region, whereas much of the research published locally in the developing world is overlooked. But it takes more than publishing good papers to become a highly cited scientist. It requires attending international meetings and introducing novel research findings in multiple scientific forums. Funding these activities, however, requires a greater proportion of research money being spent on meetings for researchers in the developing compared with the developed world.

## A Long Road Yet to Travel

The positive trends in scientific productivity in Latin America should not be misinterpreted as a reason to be unconcerned about the existing gap highlighted by Mr. Annan. There are many compelling reasons for the push to increase scientific input from the developing world (Goldemberg 1998; Annan 2003). One is that science, as a discipline, would benefit from the contributions of many disparate groups around the world, rather than being dominated by two geographic regions. Many scientific problems could be solved much more readily with the cooperation and scientific insight of scientists from developing regions. Climate change and biodiversity research, for example, urgently need the scientific input from those developing regions that are so important for these global processes. It is also critical for the developing world to promote, through research and publications, those areas of concern that are having a proportionally greater scientific and social impact upon them. There are now examples in which research on priority areas for the developing nations can actually become pioneering work in areas neglected by the research agenda of the industrialized world. This has been the case for research on renewable energy sources in Brazil (Goldemberg 1998) and biomedical sciences in Cuba (Castro Díaz-Balart 2002). These examples are important not only for those regions of the developing world, but are also in themselves scientific innovations that can greatly advance the knowledge of the rest of the world.

*Climate change and biodiversity research urgently need the scientific input from those developing regions that are so important for global processes.*

Although the evidence presented here demonstrates that there is a long way to go before developing countries contribute a more equitable share to the international scientific community, there are also reasons to be optimistic. The relative increase in the number of publications, especially when corrected for the amount of money available in research and development, demonstrates that many developing countries are heading in the right direction. The extremely high scientific productivity of many developing nations, corrected for and despite the rather limited availability of funds, suggests that increased funding to the sciences will be an excellent investment by developing nations in terms of publications as a measure of scientific output, particularly if these publications can target the journals that have the greatest impact. Although there may still be a long road to travel, we feel optimistic that the bridges mentioned by Mr. Annan are slowly being built.

## Abbreviations

GDP: gross domestic product
ISI: Institute for Scientific Information
RICYT: Red Iberoamericana de Indicadores de Ciencia y Tecnología
SCI: Science Citation Index
UNESCO: United Nations Educational
